# Encryption key distribution via chaos synchronization

**DOI:** 10.1038/srep43428

**Published:** 2017-02-24

**Authors:** Lars Keuninckx, Miguel C. Soriano, Ingo Fischer, Claudio R. Mirasso, Romain M. Nguimdo, Guy Van der Sande

**Affiliations:** 1 Vrije Universiteit Brussel (VUB), Applied Physics Research Group (APHY), Pleinlaan 2, 1050 Brussel, Belgium; 2 Instituto de Física Interdisciplinar y Sistemas Complejos, IFISC (CSIC-UIB), Campus Universitat Illes Balears, 07122 Palma de Mallorca, Spain; 3 Université libre de Bruxelles, Optique Nonlinéaire Théorique, Campus Plaine, C.P. 231, 1050 Bruxelles, Belgium

## Abstract

We present a novel encryption scheme, wherein an encryption key is generated by two distant complex nonlinear units, forced into synchronization by a chaotic driver. The concept is sufficiently generic to be implemented on either photonic, optoelectronic or electronic platforms. The method for generating the key bitstream from the chaotic signals is reconfigurable. Although derived from a deterministic process, the obtained bit series fulfill the randomness conditions as defined by the National Institute of Standards test suite. We demonstrate the feasibility of our concept on an electronic delay oscillator circuit and test the robustness against attacks using a state-of-the-art system identification method.

The development of new strategies to protect sensitive information from interception and eavesdropping has been receiving significant attention, especially in our present-day worldwide communication networks. The aim of this work is the development and implementation of a novel random key distribution system based on the concept of generalized synchronization between distant elements in large networks. Such a random key synchronization system successfully realized in photonics would have significant impact in the field of physical layer based encryption techniques, offering not only high confidentiality but also potential high-speed real-time encryption and decryption. Implemented in photonic systems, it would be fully compatible with present and future telecommunication networks. For the purpose of demonstrating the viability of the concept, we here put our focus to on an electronic system implementation.

Nowadays, confidentiality and the authenticity of information are mostly ensured through mathematical algorithms. Algorithmic key-based encryption systems usually take a digital data stream and convolute it with a given binary pattern, which we refer to as the key. The resulting encrypted binary string can then be transmitted through a public communication channel. A classic example of this type of encryption is the Vernam cipher^1^, where the recipient decodes the message using the same key-string code as used for encryption. In this case, the key is agreed via another secure channel. This algorithm has been mathematically proven to be totally secure if the key is fully random, has the same length as the message and is used only once. One-time pad cryptography is, however, not suited for secure communications between two parties who have not been able to exchange encryption keys beforehand. To circumvent this drawback, other software cryptosystems relying on asymmetric-key algorithms (public-key cryptography such as RSA) have been developed^2^. However, asymmetric algorithms use significant computational resources in comparison with their symmetric counterparts and therefore are generally not used to encrypt bulk data streams. Also, the effectiveness of these encryption techniques relies on the fact that it is computationally hard (but not impossible) to decrypt a message only knowing the public key^3^. Therefore, the growing computational power and the fact that a key is used more than once remains a latent threat for current algorithmic cryptography. Recently, although the asymmetric key algorithm itself was not broken, the Heartbleed bug in OpenSSL allowed for harvesting private keys from server communications^4^.

In order to strengthen the process of securely exchanging a private key other -hardware oriented- approaches have been proposed such as quantum cryptography. However, quantum cryptography, while secure in theory if operating at the quantum level, cannot encrypt information in real time and its key generation rate and transmission distance is limited due to noise and attenuation in the quantum channel^5^. Also, it is not compatible with standard fiber optic networks because standard telecom components such as optical amplifiers would disrupt its workings^6^. An interesting alternative electronic approach, similar to the idea of quantum entanglement but limited to wired communications, is presented by Kish^7^.

A complementary way to improve the confidentiality of an encrypted message can be realized by additionally encoding at the physical layer using chaotic carriers. Chaos based encryption systems rely on two spatially separated chaotic systems to synchronize with each other. Once the two systems are synchronized, the chaotic output of the sender can be used as the carrier in which the message is hidden as a small modulation. The receiver can extract the message by comparing the incoming signal with the synchronized one^8^. Multi-gigabit information transmission in real installed optical networks over several tens of kilometers have been demonstrated using this paradigm^9^. However, the necessity of sharing a chaotic carrier signal over a public channel reveals information on the specifics of the system used. Therefore, these chaos based communication systems offer confidentiality but cannot, for the moment, guarantee security. Such chaos based encryption schemes could augment the security by operating in a bidirectional fashion, whereby the modulating messages from both communicating parties involved perturb the shared synchronization inducing signal. Since both parties have exact a priori knowledge of their own respective modulating signal, they are able to deduce the message imposed on the carrier by the other party^9^,^10^. However, an optimized hardware solution (compatible with software methods) for confidential data transmission, possibly operating at the high bit rates that photonics offers, is currently lacking and highly desired.

## Encryption Key Distribution Via Chaos Synchronization

**Figure 1:**
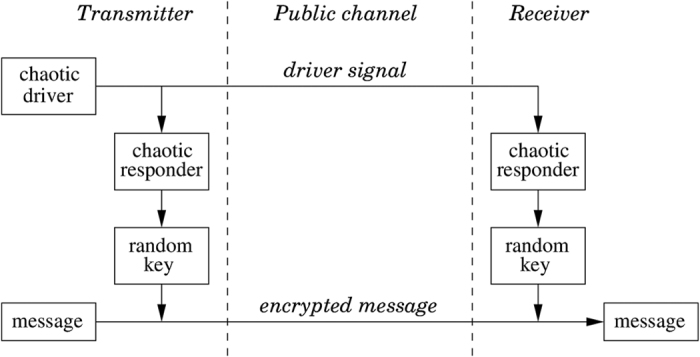
Conceptual scheme of real-time message encryption and decryption based on the novel random key synchronization concept.

The goal of our work is to demonstrate a system which can encrypt data in a new way, with a high level of security and which can be built using current off-the-shelf components. We propose a concept, built here in electronics, that later can be developed in photonics. We refer to the scheme as key distribution based on synchronized random bit generation. It relies on the synchronization between a transmitter and a distant receiver through an uncorrelated chaotic driver signal. From the synchronized chaotic signals, a random key can be distilled that would be extremely difficult to be reconstructed from the information shared in the public channel. Figure 1 shows the conceptual scheme, with a transmitter module on the left hand side and a receiver module on the right. Transmitter and receiver can communicate with each other over a public channel. The transmitter module contains an autonomous chaotic driver and a chaotic responder system, while the receiver module has a chaotic responder system identical to the chaotic responder of the sender module. Both driver and responder consist of several interacting/networked nonlinear elements. The driver generates a broadband chaotic signal, which is sent to both responders via a public channel. If both responders are practically identical, synchronization between the responders of the transmitter and receiver modules can be established through the signal of the chaotic driver. To this end, the responder systems need to react consistently to the chaotic driver, meaning that given identical inputs, regardless of their respective initial internal states, the responder states eventually synchronize to each other^11^,^12^.

If the driver and responders generate sufficiently complex dynamics (typically if these signals originate from a large network or high-dimensional nonlinear system), the generalized synchronization allows that the driver signal can have low to zero correlation and mutual information with the responder’s signal^13^. This has been proven to be the case for many other interdependence indicators^14^. From the broadband chaos at the output of the sender module’s responder, random bits can be generated by sampling the chaotic time evolution and by converting analog signals to digital. These bits will form the private key that is used to encrypt a message. The encrypted message as well as the driver’s signal are transmitted through the public channel. At the receiver module, the synchronized responder generates the same random bit sequence as was used for the encryption, allowing for immediate message decryption. Note that the proposed system, which we experimentally prove the viability of in this paper, differs significantly from standard chaos-based communications. In such systems, the message is either hidden as a low-power perturbation of the chaotic carrier or the chaotic carrier itself is used as the key. In our proposed system, an eavesdropper cannot derive information regarding the decryption key from just eavesdropping in the public channel, due to the unknown transformation that the driver signal undergoes in the responders.

This approach has several advantages compared to asymmetric key-algorithms, standard chaos-based communications and quantum key distribution systems. While the one-time pad key, that has the same length as the total message, is being generated bit per bit, the message can be encrypted in real-time, meaning without a computationally expensive operation. Because the key is derived from a chaotic signal, the same key will not be generated twice. These properties lead to a very attractive encryption protocol.

The proposed method hinges upon the identical properties of the receivers, such that we see a hardware implementation being deployed between large-volume data exchange facilities, where control over the physical access to the devices is guaranteed. The security is offered by the difficulty to access the hardware so that there is no chance to extract its dynamical signal transformation. Practically speaking, the method we present can be fully translated to the digital domain by being implemented as delay-coupled iterated maps using a field programmable gate array (FPGA) or application specific integrated circuit ASIC). The parameters of the delay coupled maps that control the dynamical behavior of the system can then be seen as a pre-shared key.

One obvious attack vector would be to duplicate the exact system that is used on the receiver’s side. However, synchronization can only be achieved when the responder system is build with devices having very similar parameters (within tolerances of a few percent or less). For an electronic implementation (as demonstrated below) the receivers could be made almost identical and truly unique by pairwise growing them side-by-side on a single wafer and/or by pairwise laser-trimming the on-chip resistors. Consequently, a brute-force attack by stepping hardware devices would be overly time consuming.

A second attack vector is a brute force one with an eavesdropper measuring the signals locally at either the sender or receiver site and reconstructing the high-dimensional, nonlinear transformation function connecting the driver’s and responder’s signals. Note that for this attack vector to succeed, physical access to a receiver is a prerequisite, that given the application site, may be easily blocked. We will discuss this issue further in the text.

Hence, to ensure high confidentiality of the message two challenging requirements remain. First, the key needs to be indistinguishable from a random bitstream. This will depend on the properties of both the chaotic driver and the responders. Second, information on the generated random key cannot be retrieved from the chaotic driver signal and its properties. Therefore, to achieve random key synchronization, we need to demonstrate chaos synchronization through a driving signal that has almost zero correlation with the responder signals. The chaotic signals should be broadband enough to support fully random bit extraction. Moreover, the system must be built in such a way that noise cannot disrupt its operation.

As seen from Fig. 1, it is a necessity that the driver signal and the encrypted message remain in synchrony. We assume that the branches of the public channel delay the driver and encrypted message signals equally, such that the signals remain synchronized in time. In an analog implementation, this requires the same group velocity over the transmission media. In a practical telecom setup, the analog driver signal is likely to be digitized first and put into numbered frames, before being transported over the same physical medium as the digital encrypted message. Thus synchronization would be guaranteed by the higher layers of the communication protocol.

## Experimental Setup

**Figure 2:**
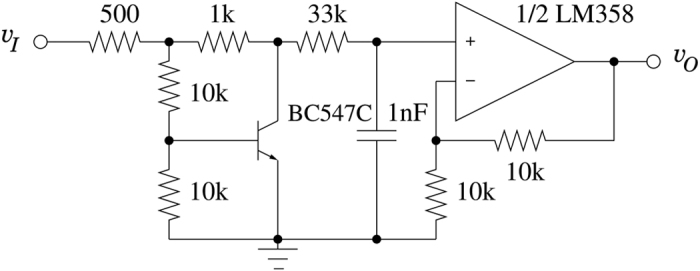
The nonlinear block (NLB).

**Figure 3:**
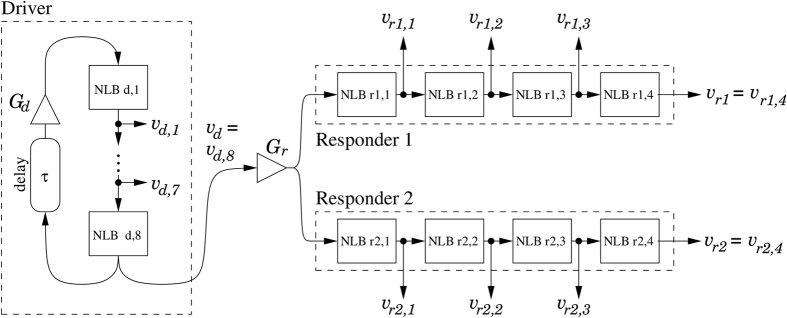
System diagram of the driver and responders. Each block labeled “NLB” contains the subcircuit of Fig. 2. Pairwise NLB blocks in the responder chains were built using matched components.

In the following, we describe the experimental electronic system that we have constructed to demonstrate the concept. The system uses several first order nonlinear blocks (NLBs) as depicted in Fig. 2. A single NLB consists of a nonlinear unimodal function, built around a bipolar transistor. The nonlinear function is followed by an RC network, acting as a low-pass filter with a characterisitc time of 33 μs, and a non-inverting × 2 amplifier. The dynamical behavior of an NLB is adequately modeled by:


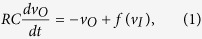


where the nonlinearity *f* is described by the Mackey-Glass^15^ function Eq. (2):


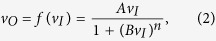


with parameters *A* = 1.99, *B* = 0.466 V^−1^, and exponent *n* = 8.38. The purpose of the amplifier is to map the input and output dynamic ranges, roughly 0 … 3 V, onto each other. It also acts as a buffer, such that NLBs can be cascaded. The resistor of the RC network is chosen much larger than the output resistance of the nonlinearity. In Fig. 3, we show a diagram of the complete system, which consists of a chaotic signal source, called the driver, and two ideally identical responder branches. The driver has eight NLBs, placed in a ring with a delay *τ* and programmable gain *G*_*d*_. Labeling *v*_*d,i*_ for *i* = 1 … 8, the output voltages of the NLBs, the driver is described by:


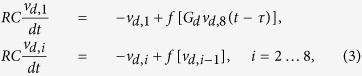


and 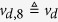 is the output of the driver. The Mackey-Glass nonlinearity with delay belongs to the class of high-dimensional dynamical systems^16^.

Because the delay and the NLBs are commutable, the driver circuit is equivalent to eight NLBs, each coupled with delay *τ*/8 in between. This is reminiscent of an eightfold Mackey-Glass system. The driver signal passes a programmable gain *G*_*r*_, and drives two responder branches, each consisting of four NLBs. The signals of the first responder branch *v*_*r*1,*i*_, *i* = 1 … 4 are described by:


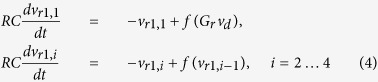


and 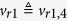. Similar equations describe the signals of the second responder branch. Much care was taken to match the corresponding components of both responders as closely as possible, in order to obtain a near identical response to the driver signal. This is achieved by using resistors with one percent tolerance, and matching the transistors and capacitors of corresponding placement in the branches. A more practical integrated circuit approach, where both responders are manufactured on a single wafer which is sliced afterwards, would yield even better matching. Note only the pairwise NLBs of the responders require matching. Within a responder, the NLBs may differ from one another, and this can even be exploited to guarantee a unique transformation. The delay and gains reside on a Digilent Nexys II field programmable gate array (FPGA) platform, which also provides storage memory for the measured signals. It is programmed to sample at *f*_*s*_ = 250 kHz. The delay line has a length of *N* = 10000 samples, corresponding to *τ* = 40 ms ≈ 1212*RC*.

## Results

**Figure 4:**
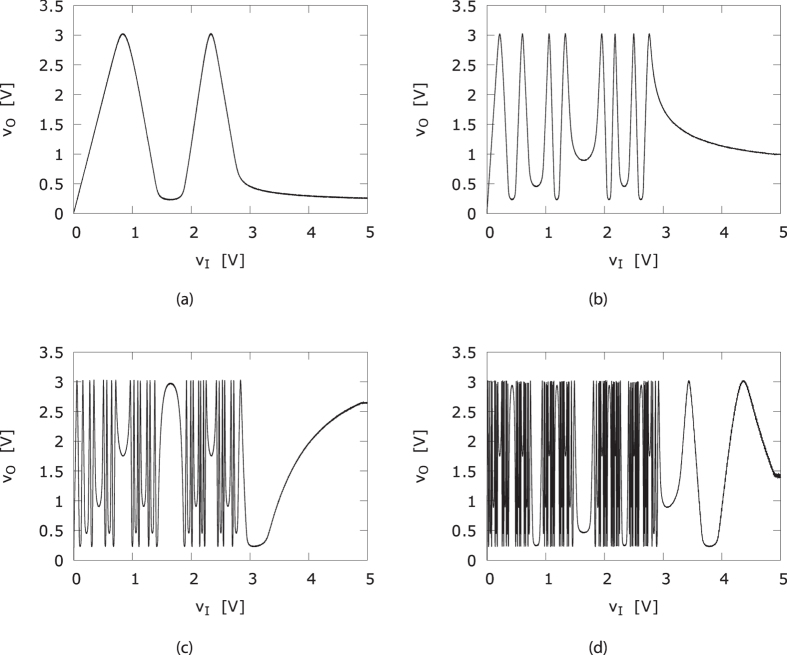
Input-output responses for (**a**) two, (**b**) four, (**c**) six and (**d**) eight cascaded NLBs when slowly scanned.

In Fig. 4, we show the nonlinear input-output characteristics for several cascaded NLBs, when scanned slowly, i.e. *v*_*O*_ = *f *^(*n*)^(*v*_*I*_). It is clear from Fig. 4, that each nonlinear transformation adds complexity to the dynamical behavior of the responder and driver signals. Intuitively, because of the unimodal character of *f*, most output values can originate from two input values, i.e. *f*^−1^(*y*) = {*x*_1_, *x*_2_}, such that *f(x*_1_) = *f(x*_2_) = *y*. Cascading *n* such (static) functions then leads to 2^*n*^ possible input values for each output value. Also, the resulting function *f*^(*n*)^ has 2^*n*−1^ local maxima, of which the abscissa, in the limit *n* → ∞, form a Cantor set.

**Figure 5:**
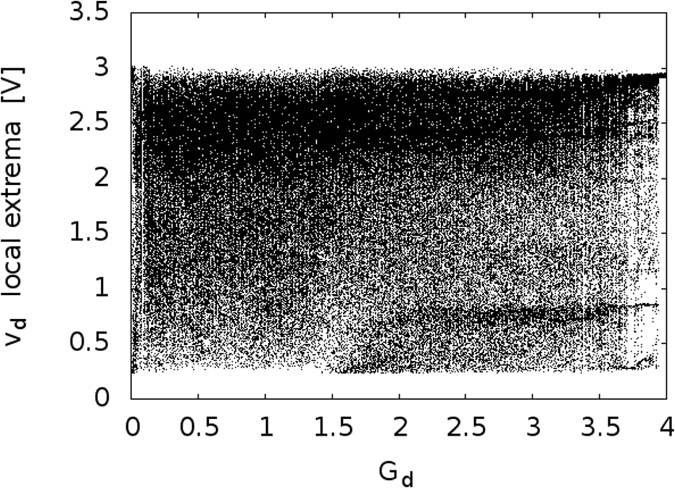
Experimentally obtained bifurcation diagram for the driver. The gain *G*_*d*_ is digitally controlled in the FPGA based delay line.

**Figure 6:**
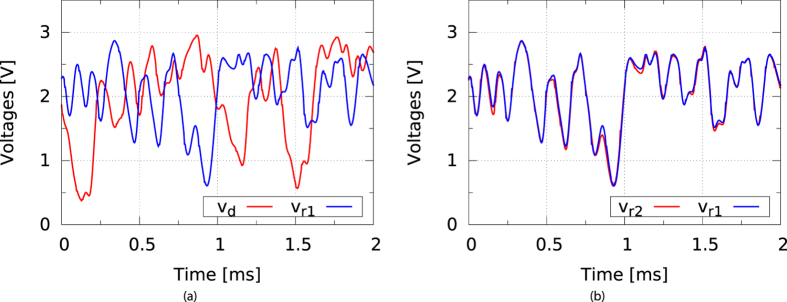
(**a**) Measured timeseries of driver (red) and responder 1 (blue) and (**b**) of responder 2 (red) and responder 1 (blue).

Figure 5 shows an experimentally obtained bifurcation diagram of the driver. For a wide range of loop gains *G*_*d*_, as programmed in the FPGA based delay line, the driver signal is clearly chaotic. We note that the driver dynamics takes several hundred delay times to reach a steady-state dynamical regime. All subsequent results are measured after this warm-up period. In turn, the responders are fully synchronized after a transient period of about 2 ms (linked to the RC time of the NLBs), when started from any initial condition. In Fig. 6, we show measured timeseries of the driver and responders. It is clear that while the responders show nearly identical signals, there is little or no resemblance between the signals of the driver and the responders. To quantize the difference between driver and responder signals, we calculate the normalized root mean square error (NRMSE):


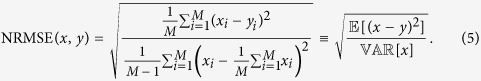


From a measurement of 2 × 10^6^ samples, we obtain: NRMSE(driver, responder1) = 1.419,

NRMSE(driver, responder2) = 1.427, NRMSE(responder1, responder2) = 0.0852, showing that the two responder signals are very much alike, while there is a large difference between the responder and driver. We further characterize the (dis)similarity of these signals by looking at the auto- and cross-correlations. For sampled real-valued signals *x(i*) and *y(i*), with *i* = −*n*, …, *n*, the time-averaged cross-correlation of a single realization of the signals is calculated as:


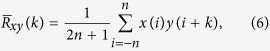


with *k* being the shift between the signals (The signals are zero-padded if an index extends outside [−*n*, …, *n*].). The means of the signals are removed, since they convey no information. This process is repeated for many (typically *m* = 50 … 100) different realizations of the signals to obtain 
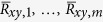
. Then these values are averaged to obtain the cross-correlation:


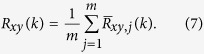


Here it is assumed that the processes from which the signals stem are wide sense stationary (WSS), so that 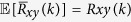. The auto-correlation of *x(i*) is calculated as above, by taking *y(i*) = *x(i*). In what follows, we normalize the correlations to:


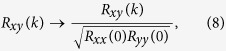


since the maximum of the auto-correlation is found at zero shift.

**Figure 7:**
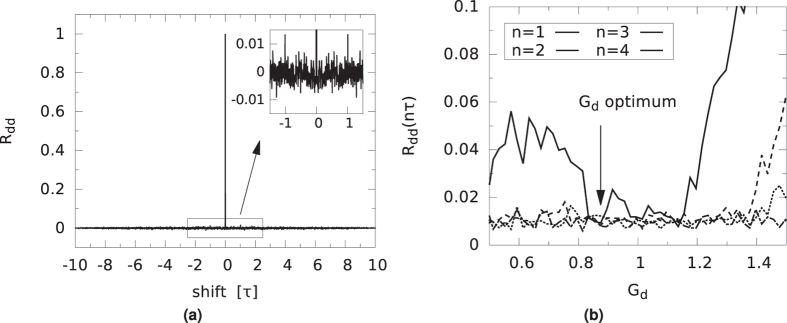
(**a**) Normalized auto-correlation function of the driver at the optimal gain *G*_*d*_ = 0.8743. (**b**) Absolute value of the normalized auto-correlation function *R*_*dd*_ of the driver at multiples of the delay time *τ*, plotted against gain *G*_*d*_.

**Figure 8:**
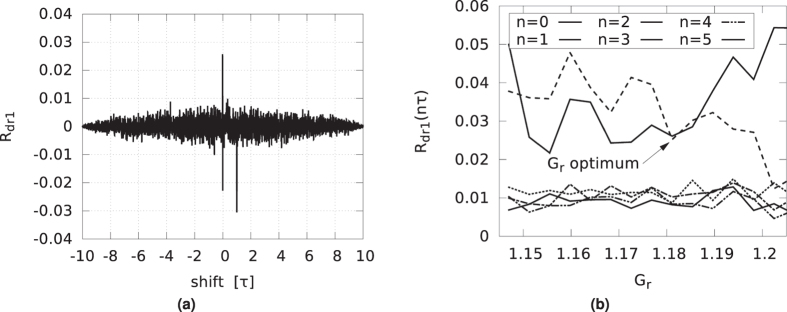
(**a**) Normalized cross-correlations of driver and responder 1, at optimal gains *G*_*d*_ and *G*_*r*_. The horizontal units are delay loop lengths *τ*. (**b**) Absolute value of the normalized cross-correlation function *R*_*dr*1_ of the driver and responder 1 at multiples of the delay time *τ*, plotted against gain *G*_*r*_.

**Figure 9:**
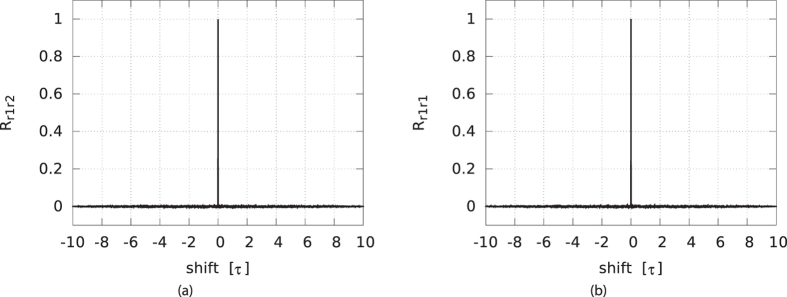
Normalized cross-correlations of the responders (**a**) and auto-correlation of responder 1 (**b**) at optimal gains *G*_*d*_ and *G*_*r*_. The horizontal units are delay loop lengths *τ*.

The normalized auto-correlation of the driver, *R*_*dd*_, has extrema at multiples of the delay *τ*. We plot the auto-correlation for the gain value of *G*_*d*_ = 0.8743 in Fig. 7a. The shift is expressed in units of *τ*. The largest residual peaks, found at ± *τ*, are below 0.015, as seen in the inset. Although derived from a deterministic system, this is close to the auto-correlation of white noise. We located this optimal gain value by plotting the magnitude of the first four peaks of the normalized auto-correlation as a function of the loop gain *G*_*d*_, in Fig. 7b. For shifts of 5*τ* and higher, these peaks were found to be negligibly small. From these results, we determined that a gain value of *G*_*d*_ = 0.8743 is optimal to minimize the sum of the absolute values of these peaks. In this way, periodic components of the driver signal are almost completely suppressed. This is important because any self-similarity in the driver signal might lead to correlations in the responder signals, which are derived from the driver. The bitstreams that are derived from these time series might then also show similarities and fail to appear random. Likewise, the cross-correlations of the driver and responder signals, *R*_*dr*1_ and *R*_*dr*2_, show peaks at or close to multiples of *τ*. In Fig. 8, we show the cross-correlation between the driver and responder 1 for gain *G*_*r*_ = 1.1811. The largest peak is below 0.03. The situation for responder 2 is similar. As before and shown in Fig. 8b this optimal gain value was determined such that the absolute sum of the peaks is as low as possible. Conversely, as shown in Fig. 9a, the responders have a near perfect correlation, as was already indicated by their low NRMSE. In Fig. 9b, the auto-correlation of responder 1, *R*_*r*1*r*1_ is shown, indicating that the noise like behavior is inherited from the driver signal. The auto-correlation of responder 2, *R*_*r*2*r*2_, is very similar to that of responder 1, and therefore is not shown.

To summarize, because the responder signals are nearly identical, the bitstreams derived thereof will also be nearly identical. The bitstream derived from the driver signal will inherit its very low long-term auto-correlations. More so, the low cross-correlation between the driver and the responders will result in nearly uncorrelated bitstreams. To be able to adequately suppress the cross-correlation between the driver and the responders, the responder branches need to have a sufficient number of nonlinear nodes. In similar experiments in photonics, where each responder consisted of only one laser, driven by a random phase light source, the residual cross-correlation was as high as 0.2 and the driver signal was noiselike, implying higher correlation within the relevant bandwidth^17^,^18^. These systems are based on synchronized semiconductor lasers. In a cascade of unidirectionally coupled semiconductor lasers the synchronization is likely to be intermittently lost in a process called bubbling^19^. In a related work, for a mutually coupled laser arrangement using zero lag synchronization, an extensive reconciliation post procedure was needed to transform the merely correlated bitstreams to truely identical bitstreams usable as key over a public channel^20^. In addition, over the last decade, a number of classical private key distribution systems have been proposed using diverse physical systems either in electronics or photonics hardware^21^,^22^,^23^.

## Bit Generation

Here, we introduce a scheme for generating bits from the driver and responder signals that we call the delayed comparison method (DCM). The method automatically delivers balanced bit series. For this method to work, it is only required that the driver and responder signals, interpreted as random processes, are wide sense stationary (WSS)^24^. If we compare two instances of such a process *X(t*) at times *t*_1_ and *t*_2_, the probability that the first measurement is smaller than the second one is:


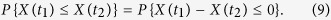


Because *X(t*) is WSS, its mean *μ*_*X*_ and variance *σ*_*X*_ are constant. Defining *Y(t*_1_, *t*_2_) = *X(t*_1_) − *X(t*_2_) as the random process of the difference, it follows that *μ*_*Y*_ = 0. Also, the probability density function of *Y* only depends on the time difference *τ* = *t*_1_ − *t*_2_ and can be written as *f*_*Y*_(*y*; *τ*). Thus:


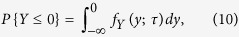


and likewise:


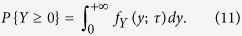


Note that *Y(t*) also has zero skew, assuming that *X(t*) has a constant median *ν*_*X*_ besides a fixed mean and variance:


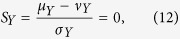


with *ν*_*Y*_ the median of *Y*. Thus the probability density function *f*_*Y*_(*y, τ*) must be symmetric around the origin. Hence *P*{*Y* ≤ 0} = *P*{*Y* ≥ 0} = 1/2, or:


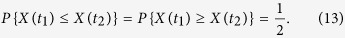


We proceed as follows to obtain the bits from the timeseries. First the timeseries *x(n*) are downsampled over a factor *r*, where *r* is chosen larger than the width of the central auto-correlation peak. This is the decorrelation step, used to avoid long successions of the same bit value. Then the resulting timeseries *x(rn*) is transformed into a series of bits *b(n*) as follows:


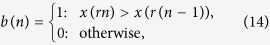


which is the deskewing step. Note that because the time series samples are discretized, there is a small probability that two samples are equal, such that Eq. (14) introduces a small bias. This can be resolved by choosing alternating values for the bits resulting from these equal samples. However, we found this to be unnecessary, and used Eq. (14) as is. Lastly, every other bit of *b(n*) is discarded, yielding the final bit series *B(n*):


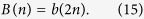


**Figure 10:**
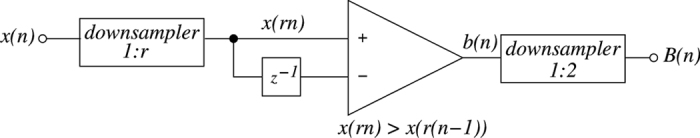
The delayed comparison method for random bits generation from a chaotic timeseries. *x(n*) is either the sampled driver or one of the responder signals.

Without this last step, one sample of the time series would be used for the generation of two bits. This repetition would eventually show up in the frequency tests to evaluate randomness. Figure 10 gives a schematic outline of the process. Since choosing a different *r*-interval results in a different bit series, it is clear that the process outlined in Fig. 10 can be applied in parallel to produce multiple bit series from one time series, thus showing an advantage in speed. For multiple intervals *r*_*i*_, the bitrate is given by:


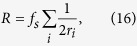


with *f*_*s*_ being the sample speed of the responder timeseries. Using *r*-intervals 81, 123, 234, 441 and 619, we obtained 22 million bits. The probability to obtain a ‘one’ for the driver and responders bitstreams are:


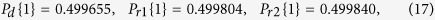


resulting in nearly maximal entropies of respectively:


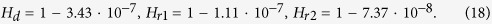


The conditional probability matrix of the resulting bit series for the responders, *P*_*r*1,*r*2_(*i, j*) = *P*{*r*1 = *i*|*r*2 = *j*} with *i, j* ∈ {0, 1}, is:


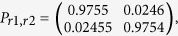


and between driver and responder 1:


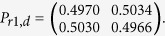


The probabilities between the driver and responder 2 are similar to those between the driver and responder 1. The mutual information between the driver and responder 1 calculates as *I*_*r*1;*d*_ = 7.3 · 10^−7^, showing that in the event an eavesdropper is able to obtain the *r*-intervals, still very little information can be obtained from the in-channel key about the responder key.

The random bits were divided in 55 sequences of 400.000 bits each. We tested these sequences with the National Institute of standards test suite for random bit streams^25^. In Table 1, we shows the results. Where a test has more than one result, the worst result is shown. The results file states that the minimum pass rate for each statistical test, with the exception of the random excursion (variant) test, is approximately 52 for a sample size of 55 binary sequences. The minimum pass rate for the random excursion (variant) test is approximately 21 for a sample size of 23 binary sequences. We conclude that the bits generated by the delay comparison method show no signs of deviation from randomness.

## Demonstration On Lena

**Figure 11:**
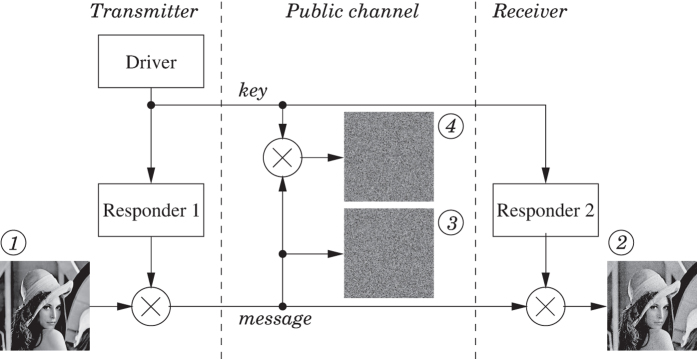
Encryption and decryption scheme, demonstrated using the Lena image as message. Full scale images are found in Fig. 12.

**Figure 12:**
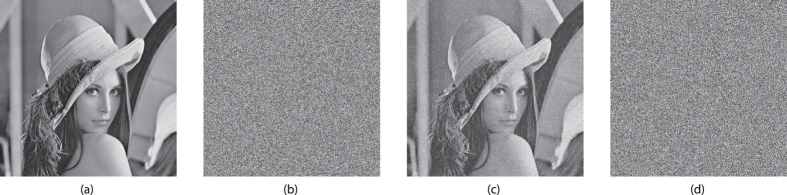
Close ups of the encrypted and decrypted messages of Fig. 11: (**a**) original unencrypted message ①, (**b**) encrypted message ③, (**c**) decrypted message at receiver end ②, (**d**) in-channel decrypted message obtained by the eavesdropper ④.

We demonstrate our encryption scheme on the “Lena” test image, as shown in Fig. 11. This grayscale version of the image consists of 512 × 512 8-bit pixels. The source image ① is encrypted with the signal of responder 1 transformed to a bitstream as described before, using an exclusive-or operation, indicated by the symbol ⊗ in the figure. This is equivalent to a modulo-2 addition. The exclusive-or based encryption is known to be vulnerable to a plaintext-attack. If the message is longer than the key and the same key is used repetitively, a known plaintext together with the encrypted message can readily reveal the key. However, in our case the key is generated on-the-fly and used only once, such that this scheme is equivalent to a Vernam cypher or one-time-pad encryption. Since the bits of the message and the key are independent random variables, the probability of a ‘1’-bit occurring in the encrypted message is:


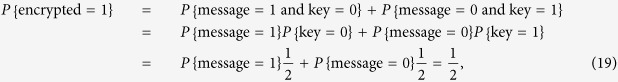


and likewise *P*{encrypted = 0} = 1/2. Thus the encrypted message is a seemingly random bitseries, showing no information about the message or the key. The encrypted message and the key are both transmitted to the receiver over the public channel. It is important that the relative phases of the key and message remain the same, once these signals reach the receiver side. In practice, this is straightforward to achieve by using established telecommunication techniques such as digitization and framing or packaging. The driver or key signal drives responder 2 in synchronization with responder 1. The bitstream derived from the signal of responder 2 is then used to decrypt the message ②, again by means of an exclusive-or operation. Some small artefacts are visible in the decrypted image, because the synchronization between the responder signals in this proof-of-concept demonstration is not perfect. Since the bit error rate is close to 0.025, an 8-bit pixel will have a probability of (1–0.025)^8^ ≈ 0.82 to be flawless. However, not all bit errors will result in visible pixel errors. Apart from extra error correction, we suggest methods for further improvement on this figure in the discussions section of this paper. Due to the unencrypted message and the responder’s bitstream being statistically independent, the encrypted message ③ is also a balanced bitstream with the same properties as the key. The message cannot be decrypted properly by an eavesdropper using the key found in the public channel, due to the uncorrelated nature of these bitstreams ④. In Fig. 12, we show the encrypted and decrypted messages again in a larger format for reference.

## A Possible Attack Using A Basis Splines Volterra Series

A possible first step in an attack on this encryption method would be to try to perform a system identification, using a set of known driver and responder signals. Note that for this method to work, an attacker needs to somehow obtain the responder signal which is not present in the channel. An up to date method for finding generalized synchronization between signals, i.e. showing how one signal is in some deterministic way derived from another, is given in ref. 26. The method is called the Functional Synchrony Model (FSM). Within the framework of FSM, a system *F* which transforms an input signal *x(t*) for *t* = 1 … *N* to an output signal *y(t*) = *F*[*x*](*t*) is modeled as a Volterra series of order *n*. Here the input *x* would be the driver signal and the output *y* the responder signal, with *F* the transformation performed by the responder system. The estimated output signal *y*_*E*_(*t*) is a sum of Volterra functionals. In ref. 26, the basis *B* consists of *M* cubic b-splines. These span a vector space of third-order piecewise polynomials with smooth nonlinearities, uniquely determined by a knot sequence *τ*_*M*_ on the memory interval [0, *M*]. Once a knot sequence is chosen, the spline functions are fully specified and can be built using the de Boor algorithm^27^. If the knots are uniformly spaced, the b-splines are simply shifted copies of each other and called cardinal b-splines. In ref. 26 both uniformly and nonuniformly spaced knots are used, the latter chosen to support maxima in the cross-correlation of the timeseries *x* and *y*.

The final model is linear with respect to the coefficients that make up the sum of the covariates. This can be solved by any number of methods. In ref. 26, elastic net regularization is used, which is further explained in ref. 28. This method seeks the coefficients 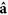 for which:


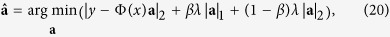


with |·|_1_ the *L*_1_-norm and |·|_2_ the *L*_2_-norm. For *β* = 0 this is a ridge regression, placing a penalty on large coefficients to avoid overfitting. Choosing *β* = 1 yields a lasso regression, resulting into a sparse set of nonzero coefficients. With a *β* parameter between one and zero, both properties can be obtained, resulting in the selection of the most important features in the data, while at the same time assuring the model generalizes well. Parameter *λ* regulates the severity of the penalty. The level of accuracy of the resulting model is measured using the NRMSE Eq. (5) and the Pearson correlation coefficient 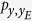:


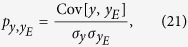


with 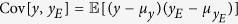, the covariance between the ideal and the estimated model output. The evaluation of the model is applied on a separate validation data set.

**Figure 13:**
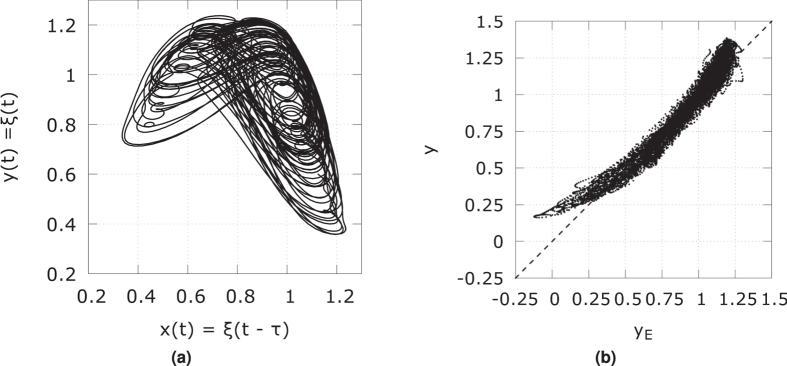
(**a**) Mackey-Glass embedding *x(t*) = *ξ(t* − *τ*) vs. *y(t*) = *ξ(t*) used for testing the FSM. (**b**) Scatter plot of desired signal *y(t*) vs. estimated signal *y*_*E*_(*t*) using a second order FSM, with parameters as in Table 2.

We implemented the above FSM scheme in a Python script, which was verified using the following Mackey-Glass system:


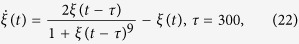


as in ref. 26, where it is shown that a single transformation *x(t*) = *ξ(t* − *τ*) → *y* = *ξ(t*) can be predicted with Pearson correlation coefficient that is close to unity. Table 2 states the parameters we used, and the resulting Pearson correlation coefficient. Figure 13a shows the input and desired output signal. Figure 13b shows a scatter plot of the FSM estimated signal vs. the desired signal, which is very much in line with what is found in ref. 26. This also indicates that a single MG-like transformation is not safe for encryption purposes.

**Figure 14:**
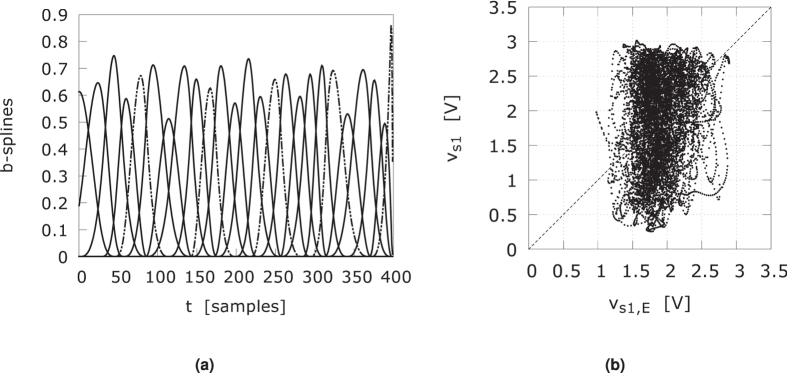
(**a**) The 25 b-splines used as basis functions for detecting synchronization between driver and responder signals. (**b**) Scatter plot of FSM estimated responder signal for four NLBs in the responder chain. The parameters are stated in Table 3.

We applied the FSM methodology, with the sampled driver signal *v*_*d*_ as input and the responder signal *v*_*r*1_ as output, in an attempt to characterize Eq. (4). The responder signals decay in about one millisecond or 250 samples at the chosen sampling rate. Therefore, we chose the spline window to be 400 samples to cover this interval. Table 3 states the parameters for the best results we could obtain, while keeping the computation time reasonable. We applied a nonuniform knot sequence, where the knots support the highest maxima of the cross-correlation of the driver and responder signals in the given window. Using a third-order approach results in 3276 covariates. The 25 b-splines are shown in Fig. 14a. As is clear from the scatter plot, Fig. 14b, the estimated responder signal *v*_*r*1,*E*_ bears little resemblance to the actual signal *v*_*r*1_. The time needed to determine the coefficients from a training time series of 30000 samples and building the testing time series of 1 million samples, was well over ten hours on an Intel dual-core laptop working at 2.4 GHz. A fourth order FSM with 25 b-splines would have 23751 covariates. We estimate that the training alone would take several days and, as suggested in ref. 26, any gain in information is easily negated by increasing the number of NLBs in the responders.

Even if the responder signal could be effectively predicted from the driver signal, an attacker would somehow still need to obtain the *r*-intervals used in the delayed comparison method to calculate the bit series. Note these intervals may be hardwired in the responders before deployment to the field and made to be even unknown to the manufacturer. We have generated the bit series resulting from the estimated responder time series, under the assumption that the attacker somehow got hold of these intervals and compared these to the bit series generated from the actual responder signal. The sample size was 14318 bits. The resulting conditional probabilities show little correlation:


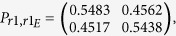


which is what we expected, given the low correlation between the estimated and real responder signal.

## Discussion

A new method for distributing encryption keys based on synchronization of driven chaotic systems has been presented. The resulting keys have passed the NIST test suite, showing no distinction from a true random bit series. The keys have the same length as the message and the encryption is done by using an exclusive-or operation. Therefore, the encryption/decryption scheme is similar to a Vernam cipher, which is proven to be unbreakable, given that the eavesdropper has no information about the key. The key is used only once and has the same length as the message.

We have demonstrated a proof-of-concept setup, based on an analog electronic system. The responder-responder synchronization is not perfect, as expected for a circuit that is made with discrete components. Nevertheless, the viability of the concept has clearly been shown. More sophisticated implementations could use delay coupled driven digital iterated maps. These can be directly implemented on a field programmable gate array or application specific integrated circuit. In previous unpublished experiments, we used six NLBs in the driver and three NLBs in each responder. However this was found to be insufficient to obtain the near-noise like auto-correlation in the driver. A fully digital implementation could easily contain even more NLBs.

Another method to obtain closely matched responder signals is to construct the analog responder circuits on a single integrated circuit wafer, which is cut after production. In this way, naturally occurring or deliberately induced process variations can be harnessed to produce truly unique systems. The driver signal can then be transmitted over a digital network, utilizing the error correction facilities already present, and converted back to analog right before entering the responder circuits. The downside of this setup would be that failure of a device on one end necessitates replacement on both ends.

A possible attack using a state-of-the-art synchronization detection method aimed at mimicking the responder system has been shown to be ineffective. In addition, the delayed comparison method for generating random bits inherently offers a second layer of safety through the unknown values and number of *r*-intervals. An attacker would need an estimate of the *r*-intervals close to the number of samples equalling the width of central peak in the driver auto-correlation. It is clear that increasing the number of NLBs and *r*-factors beyond what has been demonstrated here, leads to an increasingly complex signal transformation and thus a higher degree of confidentiality against these kind of attacks. The connection between the difference of two *r*-intervals and the resulting difference in bitstreams is still to be investigated. Note that since the *r*-values are easily reconfigured at runtime, this system could provide addressable decryption capabilities to multiple connected receivers. The concept and method presented in this manuscript is suitable for photonic implementations, compatible with current telecom infrastructures. As optical implementations, the system can e.g. be developed using electro-optical systems, which were originally proposed by Neyer and Voges^29^. A good overview of these systems is given by Larger^30^. The first application of an electro-optical system to chaos encryption was shown by Goedgebuer *et al*.^31^. This system uses a nonlinear delay feedback loop illuminated by a CW semiconductor laser. The nonlinearity is implemented through a Mach-Zehnder modulator, which is a customized integrated optics telecom device. While having good stability and controllability in real conditions, it also has architectural flexibility so that some components can be replaced to change speed, noise, efficiency etc. or to modify the architecture (additional delays, transformations etc.). An alternative setup used for generating phase chaos can also be used. In this system, the intensity modulator MZM is replaced by two other devices, namely a fast phase modulator (PM) and an imbalanced passive Mach-Zehnder interferometer (MZI), with the time imbalance longer than the characteristic time of the phase modulation. The dynamics of both systems are Ikeda-like and exhibit similar synchronization properties to the electronic circuits studied here^13^.

## Additional Information

**How to cite this article**: Keuninckx, L. *et al*. Encryption key distribution via chaos synchronization. *Sci. Rep.* **7**, 43428; doi: 10.1038/srep43428 (2017).

**Publisher's note:** Springer Nature remains neutral with regard to jurisdictional claims in published maps and institutional affiliations.

## Figures and Tables

**Table 1:** Results from the NIST randomness test suite, for the testing of 22 million bits obtained with the delayed comparison bit generation method.

Uniformity P-value	Pass Ratio	Test
0.181557	54/55	Frequency
0.025193	55/55	BlockFrequency
0.048716	54/55	CumulativeSums
0.595549	55/55	Runs
0.678686	55/55	LongestRun
0.554420	55/55	Rank
0.304126	54/55	FFT
0.678686	52/55	NonOverlappingTemplate
0.021999	55/55	OverlappingTemplate
0.366918	55/55	Universal
0.249284	55/55	ApproximateEntropy
0.186566	23/23	RandomExcursions
0.105618	23/23	RandomExcursionsVariant
0.637119	55/55	Serial
0.042808	54/55	LinearComplexity

**Table 2:** Parameters and results for the verification of our implementation of the FSM model on the Mackey-Glass system of Eq. (22).

System	Mackey-Glass, Eq. (22)
sample size	30000
sampling interval	0.1
n	2
M	28
knots interval	350, uniform
*β*	0.99
*λ*	5.5 × 10^−7^
covariates	435
nonzero coefficients	158
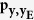	0.966
**NRMSE**	**0.2587**

**Table 3:** Parameters and results for responder system identification, using the driver and responder 1 signals.

System	4 NLBs responder, Eq. (4)
sample size	30000 fitting, 100 × 10000 testing
sampling interval	4
n	3
M	25
knots interval	400 samples, non-uniform
*β*	0.99
*λ*	1.35 × 10^−4^
covariates	3276
nonzero coefficients	3259
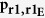	**0.1328** ± **0.0048**
**NRMSE**	**1.452**

## References

[b1] Vernam G. S. Cipher Printing Telegraph Systems For Secret Wire and Radio Telegraphic Communications. Journal of the IEEE 55, 109–115 (1926).

[b2] Rivest R. L., Shamir A. & Adleman L. A method for obtaining digital signatures and public-key cryptosystems. Commun. ACM 21, 120–126 (1978). http://doi.acm.org/10.1145/359340.359342.

[b3] Atkins D., Graff M., Lenstra A. & Leyland P. The Magic Words are Squeamish Ossifrage. In Proceedings of Asiacrypt ‘94 263–277 (Springer-Verlag, 1994).

[b4] The openSSL Heartbleed bug. http://heartbleed.com/ (2014) [Online; accessed 2-July-2015].

[b5] Bennett C. H. & Brassard G. Quantum cryptography: public key distribution and coin tossing. In *Proceedings of IEEE International Conference on Computers, Systems and Signal Processing, Bangalore, India,* (IEEE, New York, 1984).

[b6] Gerhardt I. . Full-field implementation of a perfect eavesdropper on a quantum cryptography system. Nature Commun 2 (2011). 10.1038/ncomms1348 21673670

[b7] Kish L. B. Totally secure classical communication utilizing Johnson(-like) noise and Kirchoff’s law Phys. Lett. A 352, 178–182 (2006).

[b8] Cuomo K. M., Oppenheim A. V. & Strogatz S. H. Synchronization of Lorenz-based chaotic circuits with applications to communications. IEEE TCAS II: Express Briefs 40, 626–633 (1993).

[b9] Argyris A. . Chaos-based communications at high bit rates using commercial fiber-optic links. Nature 438, 343–346 (2005). 16292256 10.1038/nature04275

[b10] Porte X., Soriano M. C., Brunner D. & Fischer I. Bidirectional private key exchange using delay-coupled semiconductor lasers. Opt. Lett. 41 (2016). 10.1364/OL.41.002871 27304310

[b11] Uchida A., McAllister R. & Roy R. Consistency of nonlinear system response to complex drive signals. Phys. Rev. Lett. 93 (2004). 10.1103/PhysRevLett.93.244102 15697817

[b12] Soriano M. C., Van der Sande G., Fischer I. & Mirasso C. R. Synchronization in simple network motifs with negligible correlation and mutual information measures. Phys. Rev. Lett. 108 (2012). 10.1103/PhysRevLett.108.134101 22540702

[b13] Van der Sande G., Soriano M. C., Fischer I. & Mirasso C. R. Dynamics, correlation scaling, and synchronization behavior in rings of delay-coupled oscillators. Phys. Rev. E 77 (2008). 10.1103/PhysRevE.77.055202 18643120

[b14] Kato H., Soriano M. C., Pereda E., Fischer I. & Mirasso C. R. Limits to detection of generalized synchronization in delay-coupled chaotic oscillators. Phys. Rev. E 88 (2013). 10.1103/PhysRevE.88.062924 24483548

[b15] Mackey M. C. & Glass L. Oscillation and chaos in physiological control systems. Science 197, 287–289 (1977). 267326 10.1126/science.267326

[b16] Farmer J. D. Chaotic attractors of an infinite-dimensional dynamical system. Physica D: Nonlinear Phenomena 4, 366–393 (1982).

[b17] Aida H. . Experiment on synchronization of semiconductor lasers by common injection of constant-amplitude random-phase light. Opt. Express 20, 11813–11829 (2012). 22714169 10.1364/OE.20.011813

[b18] Koizumi H. . Information-theoretic secure key distribution based on common random-signal induced synchronization in unidirectionally-coupled cascades of semiconductor lasers. Opt. Express 21, 17869–17893 (2013). 23938660 10.1364/OE.21.017869

[b19] Flunkert V., D’Huys O., Danckaert J., Fischer I. & Schöll E. Bubbling in delay-coupled lasers. Phys. Rev. E 79 (2009). 10.1103/PhysRevE.79.065201 19658547

[b20] Kanter I. . Synchronization of random bit generators based on coupled chaotic lasers and application to cryptography. Opt. Express 18, 18292–18302 (2010). 20721222 10.1364/OE.18.018292

[b21] Scheuer J. & Yariv A. Giant Fiber Lasers: A New Paradigm for Secure Key Distribution. Phys. Rev. Lett. 97 **(140502)** (2006). 10.1103/PhysRevLett.97.140502 17155230

[b22] Yoshimura K. . Secure Key Distribution Using Correlated Randomness in Lasers Driven by Common Random Light. Phys. Rev. Lett. 198 **(070602)** (2012). 10.1103/PhysRevLett.108.070602 22401187

[b23] Tonello A. . Secret key exchange in ultralong lasers by radiofrequency spectrum coding. Light Sci. Appl. 4 **(e276)** (2015).

[b24] Hsu H. Schaum’s outlines analog and digital communication 2nd edn. (McGraw-Hill, 2003).

[b25] Bassham L. E. . A statistical test suite for random and pseudorandom number generators for cryptographic applications. Tech. Rep., National Institute of Standards & Technology, Gaithersburg, MD, US (2010) http://csrc.nist.gov/groups/ST/toolkit/rng/index.html.

[b26] Schumacher J., Haslinger R. & Pipa G. Statistical modelling approach for detecting generalized synchronization. Phys. Rev. E 85 **5** Pt 2 (2012). 10.1103/PhysRevE.85.056215 PMC3579629 23004851

[b27] de Boor C. A practical guide to splines 1st edn. (Springer-Verlag, New York, 2001).

[b28] Zou H. & Hastie T. Regularisation and variable selection via the elastic net. Journal of the Royal Statistical Society B 67, Part 2, 301–320 (2005).

[b29] Neyer A. & Voges E. Dynamics of electrooptic bistable devices with delayed feedback IEEE Journal of Quantum Electronics 18 **(12)**, 2009–2015 (1982).

[b30] Larger L., Complexity in electro-optic delay dynamics: modelling, design and applications Philosophical Transactions of the Royal Society A 371 **(1999)** (2013). 10.1098/rsta.2012.0464 23960222

[b31] Goedgebuer J. P. . Optical communication with synchronized hyperchaos generated electrooptically IEEE Journal of Quantum Electronics 38 **(9)**, 1178–1183 (2002).

